# Unusual Case of Metastatic Gastrointestinal Adenocarcinoma to the Cervical Spine without a Detectable Primary Source in a Patient with Acquired Immunodeficiency Syndrome: A Case Report

**DOI:** 10.1155/2012/749056

**Published:** 2012-10-09

**Authors:** Paul E. Kaloostian, Marc Barry, James Fred Harrington

**Affiliations:** ^1^Department of Neurosurgery, The University of New Mexico, MSC 10 5615, Albuquerque, NM 87131-0001, USA; ^2^Department of Pathology, The University of New Mexico, MSC 10 5615, Albuquerque, NM 87131-0001, USA

## Abstract

The authors report a case of metastatic gastrointestinal adenocarcinoma to the cervical spine in a patient with acquired immunodeficiency syndrome (AIDS) being treated with antiretroviral therapy. The source of this tumor could not be identified despite a thorough evaluation. A 49-year-old male being treated for AIDS presents with worsening neck pain and left distal arm weakness. MRI demonstrated an erosive mass within the cervical four vertebral body extending through the pedicle on the left side. Patient underwent needle biopsy followed by combined anterior and posterior fusion procedures. Pathology demonstrated metastatic gastrointestinal adenocarcinoma without known primary origin. He is currently undergoing palliative radiotherapy. This is an unusual case of metastatic gastrointestinal adenocarcinoma to the cervical spine. This should be included on the differential diagnosis of spinal lesions in this patient population and may represent a unique tumor in patients with HIV/AIDS who are on immunosuppressive therapy.

## 1. Introduction

 The authors report an unusual case of symptomatic metastatic gastrointestinal adenocarcinoma without known primary tumor to the cervical spine in a patient with AIDS on chronic antiretroviral therapy.

## 2. Case Presentation

**Figure 1:**
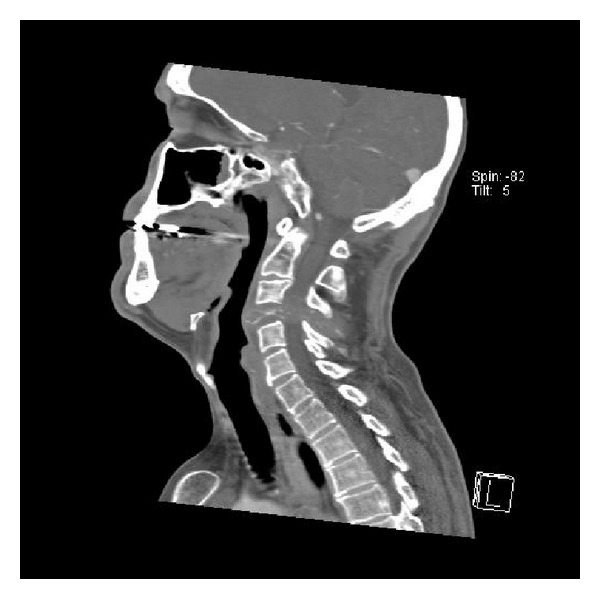
CT of the neck demonstrating erosive metastatic tumor of the C4 vertebral body.

**Figure 2:**
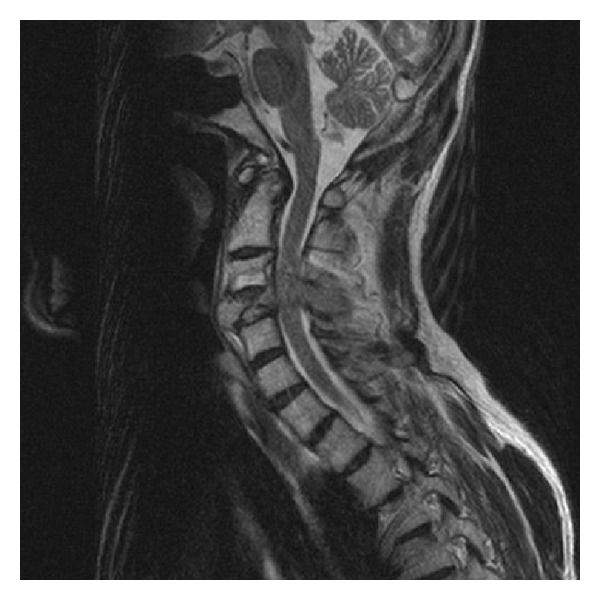
MRI of the cervical spine demonstrating erosive metastatic tumor of the C4 vertebral body.

We report the case of a 49-year-old homosexual male being treated for AIDS who presented with worsening neck pain and left distal arm and hand weakness. MRI demonstrated an erosive mass within the C4 vertebral body extending through the pedicle on the left side and causing severe spinal stenosis (Figures 1 and 2). Additionally, multiple cervical spine vertebral bodies were involved in this pathological process with the fourth cervical body being the most remarkable. PET scan, CT scan of chest/abdomen/pelvis, prior recent colonoscopy, and upper endoscopy were all performed demonstrating no obvious source. No other lesions were noted elsewhere. 

**Figure 3:**
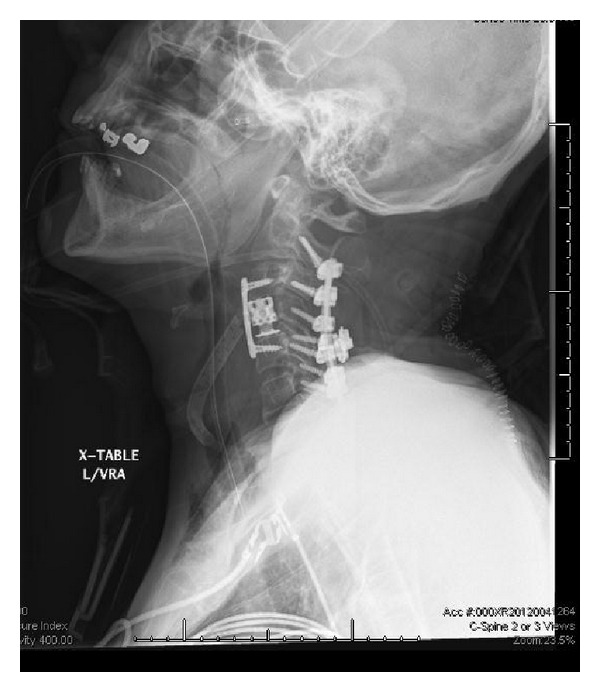
Postoperative cervical spine X rays demonstrating cervical corpectomy with placement of cage and posterior lateral mass instrumentation.

**Figure 4:**
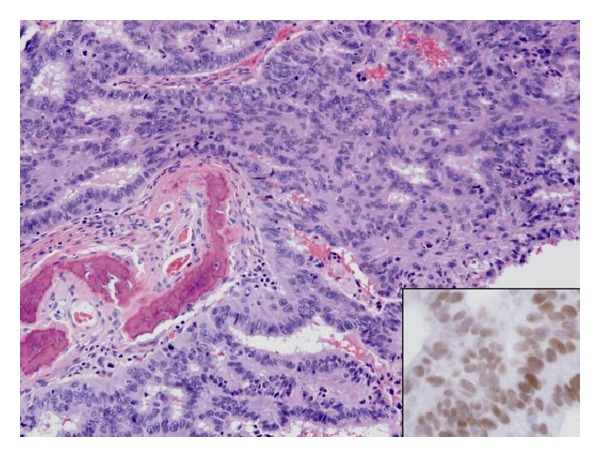
Metastatic adenocarcinoma involving trabecular bone (H&E, 200x), with (inset) immunoperoxidase staining of tumor for CDX-2 (400x).

Patient underwent needle biopsy followed by anterior cervical corpectomy and fusion and finally posterior lateral mass instrumentation and fusion (Figure 3). Pathological examination demonstrated metastatic adenocarcinoma composed of infiltrating glands and focal sheets of moderately differentiated tumor (Figure 4). Immunohistochemical staining with appropriate controls shows that the tumor cells are positive for cytokeratin 7, cytokeratin 20, and CDX-2 and are negative for TTF-1 and napsin. The morphologic and immunohistochemical findings are most consistent with tumor origin from a gastrointestinal primary tumor, in particular from an upper gastrointestinal or pancreaticobiliary primary tumor.

The patient was successfully treated for post-operative cerebrospinal fluid collection in the neck with a lumbar drain. His neurological examination returned to its baseline. His CD4 counts remained stable preoperatively and postoperatively. He is currently undergoing palliative radiotherapy with 37.5 Gy over 15 fractions to his cervical spine.

## 3. Discussion

AIDS-defining cancers, such as Kaposi's sarcoma, non-Hodgkin's lymphoma, and cervical cancer, are quite common in patients with end-stage AIDS [1–3]. Over the last few decades with the advent of antiretroviral therapy, the incidence of these cancers has increased significantly [1, 3]. Additionally, the incidence of non-AIDS-defining cancers has increased in this patient population due to the increased longevity of patients on such medications [3–5]. These include such malignancies as anal cancer, lung cancer, hepatocellular cancer, and head and neck cancers [3–5]. Over the last few decades, a mortality in mortality in this patient population has in fact been associated with these non-AIDS-defining malignancies [3]. It is hypothesized that the long-term immunosuppression, increased longevity with AIDS, and exposure to various carcinogens such as tobacco and drugs contribute to this increased incidence [3–5]. 

 Some authors have argued that there is an association between chronic AIDS and human immunodeficiency syndrome (HIV) infection and the occurrence of colonic malignancy [4]. Studies have suggested that young age and advanced stage at time of diagnosis carry the greatest weight in classifying a poorer prognosis [3, 4]. It is well known that recipients of organ transplants are similarly known to have an increased incidence of cancer, believed to be related to the length of immunosuppressive drugs use to prevent rejection [3, 4].

About 10% of all cancer patients develop metastases to the spine [4, 5]. Among immunocompetent adult patients with cancer, 60% of these spinal metastases are either from the breast, lung, or prostate [4, 5]. Renal and gastrointestinal cancers each account for 5% of spinal metastases [4, 5]. In patients with AIDS, this differential diagnosis is quite different. Pathology may include non-Hodgkin's lymphoma, Kaposi's sarcoma, metastasis, and infection. To add to this complexity, unknown primary tumors in patients with clearly biopsy-proven metastatic disease are quite rare [1, 2, 6]. This incidence is in the range of 0.5%–38% [7, 8]. In these patients in whom a primary source could not be identified, antemortem studies have demonstrated definitive pathological diagnosis in 31% of cases, with a range being 7% to 88% in studies looking at patients with spinal metastatic disease [8]. In one study, lung cancer turned out to be the most common cancer found in these patients with initially an unknown primary site 56% of the time [9]. Interestingly, this study also demonstrated a significant increase in survival in patients with noncervical spinal disease as compared to those with isolated cervical metastatic disease [9]. Patients with extraspinal disease at presentation also had poorer survival compared with those who did not, hypothesized to be due to increased tumor burden [9]. 

Ravalli et al. noted in their seminal report three patients with HIV in less than one year who developed gastrointestinal carcinoma and suggested an increased frequency in this population [5]. Gastrointestinal metastasis to the spine is unusually rare. Reports of esophageal cancer, carcinoid tumor in a patient with multiple endocrine neoplasia, rectal cancer, and colonic adenocarcinoma have been reported [5, 10]. Other than Ravalli et al., a review of the literature noted no reports of patients with AIDS/HIV and associated gastrointestinal adenocarcinoma of unknown primary tumor despite full workup. 

This case stimulates interest in a possible association between AIDS/HIV, long-term antiretroviral therapy, and metastatic gastrointestinal adenocarcinoma without a clear primary site. We wonder if this particular metastatic gastrointestinal tumor is a unique tumor of the gastrointestinal system that is associated with chronic HIV/AIDS or chronic immunotherapy. In conclusion, this pathology must be kept on the differential diagnosis list in this patient population and further cases must be documented to clearly confirm this association.
